# Myocardial Infarction in a 19-year-old with a History of Kawasaki Disease: A Case Report

**DOI:** 10.5811/cpcem.50676

**Published:** 2026-04-21

**Authors:** Chad R. Sethman, Jessica R Sethman, Bradley M End

**Affiliations:** * West Virginia University, Department of Microbiology, Immunology and Cell Biology, Morgantown, West Virginia; † West Virginia University, Department of Emergency Medicine, Morgantown, West Virginia; ‡ West Virginia University, Department of Emergency Medicine, Department of Medical Education, Morgantown, West Virginia

**Keywords:** Kawasaki disease, coronary artery aneurysm, acute coronary syndrome, electrocardiogram, case report

## Abstract

**Introduction:**

Kawasaki disease is a vasculitis most commonly affecting children under five years of age but can also occur in older children and adults. When not sufficiently treated, Kawasaki disease can lead to cardiac complications such as myocarditis and coronary artery aneurysms, with aneurysms being the most serious long-term complication as it poses a risk for acute coronary syndrome.

**Case Report:**

A 19-year-old with remote history of Kawasaki disease presented to the emergency department with chest pain, diaphoresis, and emesis after being struck in the chest by another player during a basketball game. Despite his young age and reported mild musculoskeletal trauma, an electrocardiogram and troponin were ordered. Electrocardiogram findings were concerning for ischemia, and troponin was elevated, confirming myocardial infarction and prompting a cardiology consult. Urgent percutaneous coronary intervention of the occluded aneurysm with balloon angioplasty resulted in significantly improved distal blood flow.

**Conclusions:**

Kawasaki disease is widely recognized as a cause of cardiac complications in childhood, but the risk does not end there. Adults with a history of childhood Kawasaki disease remain at risk for complications of coronary artery aneurysm, even when they received appropriate medical treatment. Thrombosis or dissection of a coronary artery aneurysm can lead to acute coronary syndrome in otherwise healthy individuals. Therefore, emergency physicians must maintain a high level of suspicion for cardiac complications in both children and adults with a history of Kawasaki disease. In this case, prompt diagnosis and intervention were essential to achieving the best possible outcome.

## INTRODUCTION

In 1967, a report by Tomisaku Kawasaki described a distinct type of vasculitis in 50 infants in Japan.^1^ Now commonly known as Kawasaki disease, the incidence of this disease has been steadily increasing and has now emerged as the most common cause of acquired heart disease in children in developed countries.^2^–^4^ The exact etiology is unknown; however, inflammatory mediators in the blood lead to significant vascular inflammation that can cause the vessel walls to thicken, narrow, or weaken.^5^,^6^ Furthermore, the vasculitis associated with Kawasaki disease tends to localize to coronary arteries and can lead to cardiac complications such as myocarditis and coronary artery aneurysms, with aneurysms being the most serious long-term complication.^7^ Thrombus formation within the coronary artery aneurysm can result from a combination of abnormal blood flow and platelet aggregation at the damaged endothelium leading to cardiac ischemia and myocardial infarction (MI).^8^

Various treatment options are available to mitigate the severity, duration and sequelae of Kawasaki disease. The primary objectives in treatment include the reduction of systemic vascular inflammation and inhibition of thrombosis formation. Therefore, treatment typically involves the administration of a single intravenous immunoglobulin infusion and low-dose acetylsalicylic acid.^9^ More aggressive treatments include antiplatelet and anticoagulant therapies such as clopidogrel, enoxaparin, and alteplase for higher risk patients and those who develop large aneurysms.^3^,^9^,^10^

The incidence of Kawasaki disease can be 25% or higher in untreated or delayed-treatment cases compared to an estimated 4% in those who are treated in a timely manner.^7^,^11^ Acute coronary syndrome most commonly occurs within the first two years following Kawasaki disease diagnosis, but cases have also been reported in adults long after the initial diagnosis especially in individuals with persistent coronary artery aneurysm.^12^–^14^ Therefore, vigilant monitoring of patients for aneurysm formation during the acute phase of illness, along with lifelong assessment for thrombotic occlusion, is crucial for effective treatment and long-term management.^8^,^15^

## CASE REPORT

A 19-year-old male college student presented to the emergency department (ED) for chest pain after being struck in the chest by another player during a basketball game. He reported experiencing nausea and vomiting for two hours with associated mild dyspnea, diaphoresis, and light-headedness. He reported having had Kawasaki disease at age five, but it was undetermined whether he had treatment at that time. He did have a known right coronary artery aneurysm for which he routinely saw cardiology in his hometown and was prescribed daily acetylsalicylic acid and clopidogrel to reduce the risk of thrombus formation. Echocardiogram and cardiac stress tests were performed one month prior to his ED visit, which showed retained ejection fraction and no signs of coronary artery occlusion at that time, but images were not available for review at the time of his encounter.

On examination he was well appearing, resting comfortably, and conversing with an accompanying basketball player. Vital signs were as follows: blood pressure, 92/55 millimeters of mercury; heart rate, 62 beats per minute; respiratory rate, 18 breaths per minute; temperature, 36.2 °C, and oxygen saturation 95% on room air. Auscultation of the heart demonstrated a regular rhythm with no murmurs, gallops, or rubs. He had no tachypnea, respiratory distress, or adventitious lung sounds.

**Image 1:**
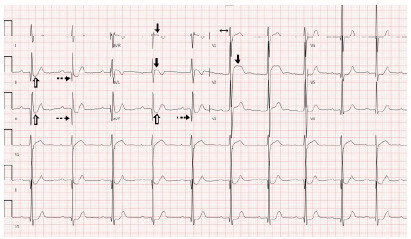
Electrocardiogram upon emergency department arrival of a young patient with chest pain that demonstrates sinus rhythm with a first-degree atrioventricular block (double-sided arrow). Q waves (dashed arrows) and ST-segment depressions (open arrows) are observed in the inferior leads (II, III, and aVF). ST-segment elevations (solid arrows) are present in V2, aVR, and aVL.

An electrocardiogram (ECG) was performed (Image 1) and revealed sinus rhythm with first-degree atrioventricular block, Q waves, and ST-segment depressions in the inferior leads as well as nonspecific ST-segment elevations in noncontiguous leads. The patient’s cardiac troponin was 328 nanograms per liter (ng/L) (reference range: < 30 ng/L). Repeat troponin was not performed prior to intervention. His white blood cell count was 12,100 per liter (L) (< 11,000/L), and his basic metabolic panel was within normal limits. There was no acute cardiopulmonary process identified on chest radiograph. Given the heightened level of concern due to the patient’s history, continued chest pain, ischemic morphology of the ST-segment elevation in V2 with other ECG changes, and elevated troponin, cardiology was consulted and recommended urgent cardiac catheterization despite the patient not having met ST-segment elevation myocardial infarction (STEMI) criteria.

*CPC-EM Capsule*

What do we already know about this clinical entity?

*Kawasaki disease, a vasculitis most commonly affecting children, can lead to coronary artery aneurysms and subsequent thrombus formation when left untreated*.

What makes this presentation of disease reportable?

*An otherwise healthy 19-year-old with a history of Kawasaki disease presented with chest pain following musculoskeletal trauma. Cardiac assessment revealed myocardial infarction*.

What is the major learning point?

*Emergency physicians should maintain a heightened level of suspicion for acute coronary syndrome in patients with a history of Kawasaki disease*.

How might this improve emergency medicine practice?

*This case highlights the long-term complications of Kawasaki disease, and the need for timely cardiac assessment and intervention*.

**Image 2:**
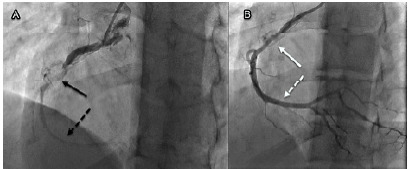
Cardiac catheterization images pre/post angioplasty of young man with history of Kawasaki disease: A) Initial angiography image revealing a visible cardiac artery aneurysm (solid black arrow) and diminutive distal flow due to occlusion/thrombus (dashed black arrow); and B) angiography following percutaneous coronary intervention with balloon angioplasty showing the aneurysm (solid white arrow) with significantly improved distal flow (dashed white arrow).

The catheterization revealed an acute near-total thrombotic occlusion of the aneurysm in the proximal portion of the right coronary artery (Image 2A). Balloon angioplasty and thrombectomy were performed at the time of catheterization (Image 2B), followed by stent placement.

The patient had an uncomplicated clinical course following percutaneous coronary intervention. Subsequent echocardiogram showed mildly depressed left ventricular function and moderately depressed right ventricular function with an ejection fraction of 45%. The patient was discharged in stable condition on postoperative day three with the diagnosis of non-STEMI (NSTEMI). It was recommended that he continue taking 81 mg acetylsalicylic acid once daily as an outpatient and begin taking 20 mg rivaroxaban once daily. The previously prescribed clopidogrel was discontinued.

## DISCUSSION

Although this patient was being managed by cardiology to prevent complication of a known right coronary artery aneurysm from childhood Kawasaki disease he still ultimately developed a near-complete occlusion of the right coronary artery leading to acute MI. Treatment principles for MI in Kawasaki disease patients are derived from the same guidelines established for the adult population with atherosclerotic coronary artery disease. Accordingly, indications for urgent cardiac catheterization in Kawasaki disease patients mirror those recommendations.^3^ In this case, the presence of Q waves and ST-segment depressions in leads II, III, and aVF indicated an age-undetermined infarction. The ST-segment elevations observed in V2, aVR, and aVL are considered nonspecific as there are no two contiguous leads of elevation.

Although this patient did not technically meet the criteria for STEMI, he did fulfill the diagnostic criteria for NSTEMI and cardiology determined that expedited percutaneous intervention was necessary. Further workup revealed a newly decreased ejection fraction, validating the need for urgent intervention. Given these findings, it is imperative that emergency physicians maintain a heightened level of suspicion for acute coronary syndrome in patients with a history of Kawasaki disease.

## CONCLUSION

Because acute coronary syndrome is infrequently detected in young adults presenting with chest pain, emergency physicians may be less inclined to expand the cardiac workup beyond an ECG. While it is widely understood that Kawasaki disease can cause cardiac complications in childhood, it is important to recognize that even with appropriate medical therapy, these patients continue to carry the risk of coronary artery aneurysm and, thus, acute coronary syndrome, throughout adulthood. These late complications often present acutely, and emergency physicians are frequently the first point of contact when they do. Therefore, if a patient with a history of Kawasaki disease in childhood presents for chest pain, emergency physicians should strongly consider a full cardiac evaluation to rule out acute coronary syndrome even if the patient appears well and the history of present illness suggests a potential noncardiac etiology such as, in this case, musculoskeletal trauma.
